# Safety of Drugs Used during the First Wave of COVID-19: A Hospital-Registry-Based Study

**DOI:** 10.3390/diagnostics12071612

**Published:** 2022-07-01

**Authors:** Cristina Aguilera, Immaculada Danés, Elena Guillén, Alba Vimes, Montserrat Bosch, Gloria Cereza, Adrián Sánchez-Montalvá, Isabel Campos-Varela, Marta Miarons, Jaume Mestre-Torres, Antònia Agustí

**Affiliations:** 1 Clinical Pharmacology Service, Vall d’Hebron Hospital Universitari, Vall d’Hebron Barcelona Hospital Campus, 08035 Barcelona, Spain; craguile@vhebron.net (C.A.); eguillen@vhebron.net (E.G.); avimes@vhebron.net (A.V.); mobosch@vhebron.net (M.B.); aagusti@vhebron.net (A.A.); 2 Department of Pharmacology, Therapeutics and Toxicology, Universitat Autònoma de Barcelona, Bellaterra, 08193 Barcelona, Spain; gcg@icf.uab.cat; 3 Immunomediated Diseases and Innovative Therapies Group, Vall d’Hebron Research Institute, 08035 Barcelona, Spain; 4 Catalan Institute of Pharmacology Foundation, Vall d’Hebron Hospital Universitari, 08035 Barcelona, Spain; 5 International Health and Tuberculosis Unit, Infectious Diseases Department, National Referral Centre for Tropical Diseases, Vall d’Hebron Hospital Universitari, Vall d’Hebron Barcelona Hospital Campus, 08035 Barcelona, Spain; adsanchez@vhebron.net; 6 Liver Unit, Vall d’Hebron Institut de Recerca (VHIR), Vall d’Hebron Hospital Universitari, Vall d’Hebron Barcelona Hospital Campus, Universitat Autònoma de Barcelona, 08035 Barcelona, Spain; icampos@vhebron.net; 7 Centro de Investigación Biomédica en Red de Enfermedades Hepáticas y Digestivas (CIBERehd), Instituto de Salud Carlos III, 28220 Madrid, Spain; 8 Pharmacy Department, Vall d’Hebron Hospital Universitari, Vall d’Hebron Barcelona Hospital Campus, 08035 Barcelona, Spain; mmiarons@vhebron.net; 9 Internal Medicine Service, Vall d’Hebron Hospital Universitari, Vall d’Hebron Barcelona Hospital Campus, 08035 Barcelona, Spain; jmestretorres@gmail.com

**Keywords:** adverse drug reactions, off-label use, registries, COVID-19, lopinavir/ritonavir, hydroxychloroquine

## Abstract

The emergency of the coronavirus disease 2019 (COVID-19) pandemic led to the off-label use of drugs without data on their toxicity profiles in patients with COVID-19, or on their concomitant use. Patients included in the COVID-19 Patient Registry of a tertiary hospital during the first wave were analyzed to evaluate the adverse drug reactions (ADRs) with the selected treatments. Twenty-one percent of patients (197 out of 933) had at least one ADR, with a total of 240 ADRs. Patients with ADRs were more commonly treated with multiple drugs for COVID-19 infection than patients without ADRs (*p* < 0.001). They were younger (median 62 years vs. 70.1 years; *p* < 0.001) and took less medication regularly (69.5% vs. 75.7%; *p* = 0.031). The most frequent ADRs were gastrointestinal (67.1%), hepatobiliary (10.8%), and cardiac disorders (3.3%). Drugs more frequently involved included lopinavir/ritonavir (82.2%), hydroxychloroquine (72.1%), and azithromycin (66.5%). Although most ADRs recovered without sequelae, fatal cases were described, even though the role of the disease could not be completely ruled out. In similar situations, efforts should be made to use the drugs in the context of clinical trials, and to limit off-label use to those drugs with a better benefit/risk profile in specific situations and for patients at high risk of poor disease prognosis.

## 1. Introduction

Severe acute respiratory syndrome coronavirus 2 (SARS-CoV-2) was identified in early 2020, spreading rapidly worldwide and acquiring pandemic status in March 2020. No specific treatments were available, although a large number of clinical trials quickly started, assessing different treatment options and prevention modalities. The emergency of the situation and the lack of evidence-based information led, in clinical practice, to the off-label use of many treatments that have been on the market for a long time, such as chloroquine, hydroxychloroquine, lopinavir/ritonavir, azithromycin, ceftriaxone, and interferons [1,2,3]. In Spain, these medicines were used in accordance with Royal Decree 1015/2009 of 19 June, which regulates the availability of medicines in special situations [4].

Although the toxicity profile of these drugs is relatively well known in approved indications from clinical trials and post-marketing experience, caution should be exercised when using them for a new disease and in combination, due to the high potential for drug interactions [5]. One of the main concerns is that several of these drugs (i.e., chloroquine, hydroxychloroquine, lopinavir/ritonavir, azithromycin) may prolong the QTc interval and increase the risk of ventricular arrhythmias and drug-induced sudden death. Although in absolute terms the incidence of cardiac events related to each of them is low—especially in short-term treatments—the risk is expected to increase when they are used in combination (pharmacodynamic interaction) or in the presence of other risk factors [6,7,8,9,10,11]. Furthermore, their use in patients with a still-unknown pathology (e.g., coronavirus disease 2019 (COVID-19)) could be associated with a different adverse drug reaction (ADR) profile [12].

A prevalence of ADRs of 37.8% was described in 217 patients admitted to the First Hospital of Changsha, in China, over a period of one month. Gastrointestinal and hepatic disorders predominated; the drugs most commonly involved were lopinavir/ritonavir and umifenovir, and the length of hospital stay (OR 2.02, *p* = 0.04), number of drugs used (OR 3.17, *p* = 0.001), and presence of underlying basic diseases (OR 2.07, *p* = 0.04) were independent risk factors for ADRs [13].

The aim of this study was to assess the frequency, type, and main characteristics of ADRs with the drugs used to treat COVID-19 in a third-level university hospital, and to analyze possible risk factors associated with ADRs.

## 2. Materials and Methods

A retrospective, observational, hospital-based, single-center study was carried out using data from a specific registry of inpatients affected by COVID-19 at the Vall d’Hebron Hospital Universitari, in Barcelona, Spain—a 1100-bed public, university, tertiary hospital in Barcelona. At the peak of the pandemic, about 650 beds were occupied by COVID-19 patients.

The registry included adult patients who were admitted to any in-hospital department. Data on demographic characteristics, toxic habits, comorbidities, usual medications, clinical signs and symptoms, severity of COVID-19, treatment, complications, and outcomes were recorded. Information about possible ADRs during treatment and their outcomes was also included in this registry. All of the registered information was collected manually by trained clinicians from the electronic medical records of hospitalized patients diagnosed with COVID-19. For this study, patients registered between 1 March and 15 May 2020 were selected, and those for whom an ADR related to drugs used to treat COVID-19 was suspected were analyzed and compared to the rest of the patients. To guarantee the data quality of the records and avoid errors, limits were placed on the values of some variables, and a queries system was used.

ADRs were classified according to the Medical Dictionary for Regulatory Activities MedDRA^®^ [14], and drugs were classified according to the 2020 Anatomical Therapeutic Chemical (ATC) classification system [15]. The plausibility of the association between ADRs and drugs was analyzed and discussed according to the Spanish Pharmacovigilance System algorithm, which takes into account the temporal sequence, prior knowledge of the ADR, drug withdrawal effects, drug re-exposure effects (if applicable), and possible alternative causes of symptoms [16]. When information to properly assess the causality of the drug(s) was lacking, the patient’s electronic medical records were reviewed. According to previous knowledge, ADRs were classified as well-known ADRs (if they were described in the product labelling), known from anecdotal reports, and unknown. The seriousness of the ADRs was classified in accordance with the European Union’s criteria. ADRs were considered serious when they were life-threatening or resulted in death, required hospitalization or prolonged an existing hospitalization, resulted in persisting disability, or were classified as an important medical event. The remaining cases were classified as non-serious [17]. Identified suspected ADRs were reported to the Spanish Pharmacovigilance System.

Categorical variables are expressed as frequencies and percentages, and numerical variables are expressed as medians and interquartile ranges (IQRs). Chi-squared or Fisher tests were applied for comparison of categorical characteristics, and the Mann–Whitney test was used for continuous ones. Trend tests were used to analyze the relationship between the number of drugs administered (ordinal variable) and the reporting of ADRs. Significance was set at a level of 0.05, and was two-tailed. The statistical analysis was performed using the SAS^®^ 9.4 statistical package (SAS Institute Inc., Cary, NC, USA).

The study was conducted according to international ethical recommendations, and was approved by the local Research Ethics Committee, following the national directives related to post-authorization studies (protocol code VDH-2020-01, date of approval 5 June 2020).

## 3. Results

A total of 933 patients was included. Their median age was 67.7 years (IQR 52.1–78.8), and 54.3% were men. Obesity (body mass index ≥ 30 kg/m^2^) was present in 19.0% of patients, and 4.2% declared themselves to be active smokers. Intensive care unit admission was required in 12.6% of patients, and median oxygen saturation was 96 (IQR 93–98). The principal comorbidities were hypertension (47.8%), chronic respiratory disease (20.5%), and diabetes (19.8%). Seven hundred and six patients (75.7%) were receiving chronic medication, the most common being proton-pump inhibitors (34.6%), statins (26.5%), analgesics such as acetaminophen (15.2%), and ACE inhibitors (14.3%). Regarding COVID-19 treatment, 75.0% of patients received between 3 and 5 drugs, the most frequent being hydroxychloroquine (90.0% of patients), azithromycin (87.2%), ceftriaxone (71.1%), lopinavir/ritonavir (67.3%), and tocilizumab (18.6%).

One hundred and ninety-seven patients (21.1%) experienced at least one ADR, with a total of 240 ADRs. The patients’ baseline characteristics are detailed in Table 1. Compared to patients without ADRs, those with ADRs were younger (median 62 years vs. 70.1; *p* < 0.001), were taking less chronic medication (69.5% vs. 75.7%; *p* = 0.031), and some specific comorbidities (i.e., hypertension, CNS diseases, or moderate/severe cognitive impairment) were significantly less common. As shown in Table 2, patients with ADRs were more commonly treated with multiple drugs for COVID-19 infection than patients without ADRs (*p* < 0.001).

Table 3 shows that the most common ADRs were gastrointestinal (67.1%) and hepatobiliary disorders (10.8%), followed by cardiac disorders (3.3%) and abnormal investigations consisting of electrocardiogram QT prolongation (2.9%). The drugs most frequently involved (i.e., considered suspicious of causing or contributing to ADRs) were lopinavir/ritonavir (82.2% of patients with ADRs), hydroxychloroquine (72.1%), and azithromycin (66.5%), although in some cases a role of the disease could not be excluded. Treatment with levofloxacin, lopinavir/ritonavir, or interferon beta-1b was related to one or more ADRs in >20% of treated patients (Table 4). Patients treated with hydroxychloroquine, lopinavir/ritonavir, or tocilizumab were significantly younger than those not treated with these drugs (lower median age), while patients treated with ceftriaxone or darunavir/cobicistat were older, on average. Patients with arterial hypertension were less commonly treated with lopinavir/ritonavir or tocilizumab, and more commonly treated with darunavir/cobicistat or ceftriaxone (Table 5 and Supplementary Figures S1 and S2).

Diarrhea was the most frequent ADR, representing nearly half of all ADRs. Other ADRs that accounted for at least five cases were nausea, vomiting, different types of liver damage (e.g., hyperbilirubinemia, mixed liver injury, cytolysis), and QT prolongation. ADRs and suspicious drugs considered to be possibly involved in those ADRs are detailed in Table 6 and Supplementary Table S1.

In all suspected ADRs, there was a temporal relationship between the onset of signs or symptoms and the initiation of treatment with suspected drugs. ADRs were previously known for at least one of the drugs considered as suspicious except in four ADRs: two cases of bradycardia with a normal QTc interval in patients treated with azithromycin, hydroxychloroquine, and lopinavir/ritonavir (plus tocilizumab in one case); one case of atrial fibrillation with azithromycin, ceftriaxone, darunavir/cobicistat, and hydroxychloroquine; and a fall experienced in a patient treated with azithromycin, ceftriaxone, interferon beta-1b, and lopinavir/ritonavir. It should be mentioned that this fall was the consequence of other known ADRs (agitation and confusion) in the same patient. Re-exposure was not available for any case. Drug withdrawal resulted in recovery without sequelae in 87.9% of ADRs (211), and death in 4.2% (10 ADRs in 6 patients); 9 ADRs (3.7%) had not been resolved when the study was completed, and in 10 ADRs (4.2%) the outcome was unknown. Alternative causes were difficult to assess given the novelty of the disease. The possibility that COVID-19 itself could explain some of the signs or symptoms registered as ADRs was considered in many cases, including cases of well-known ADRs (e.g., diarrhea in patients receiving lopinavir/ritonavir), when the temporary relationship and course after drug withdrawal was not completely suggestive, as well as in some cases of hepatitis.

Seventeen ADRs (7.1%) were serious, and affected 14 patients. These ADRs corresponded to four cases of sudden death in patients with multiple treatments, single cases of different cardiac disorders (i.e., atrial fibrillation, bradycardia, hypotension with tachycardia and cardiac arrest, atrial flutter with cardiogenic shock, and electrocardiogram QTc prolongation), hepatobiliary disorders (i.e., one case of cholestatic hepatitis and one of mixed liver injury), and others (i.e., one case of large intestine perforation, one septic shock and one chest wall hematoma). More specifically, there were six cases with fatal outcomes: four patients suffered sudden death (three during treatment with lopinavir/ritonavir, hydroxychloroquine, and other drugs (i.e., azithromycin, levofloxacin, quetiapine, and/or risperidone); and another after registering a long QT interval while on treatment with azithromycin, hydroxychloroquine, metoclopramide, propofol, and salbutamol), and two fatal cases were due to cardiac complications after tachyarrhythmia when on treatment with hydroxychloroquine and azithromycin in one case, and with hydroxychloroquine, digoxin, nitroglycerin, and metamizole in the other case.

## 4. Discussion

This is the first published study using a specific clinical registry of patients affected by COVID-19 in a hospital with complete and easily retrievable information, including data on suspected ADRs. One out of five patients admitted to our hospital during the first wave of the pandemic had at least one ADR possibly related to administered treatments, with gastrointestinal (67.1%), hepatobiliary (10.8%), and cardiac disorders (3.3%) being the most frequent, followed by electrocardiogram QT prolongation (2.9%). Compared to patients without ADRs, patients with ADRs were younger, took less medication regularly, were less commonly affected by some comorbidities (i.e., hypertension, CNS diseases, or moderate/severe cognitive impairment), and were more frequently exposed to multiple treatments for COVID-19 or related complications. The drugs most frequently considered to be involved in ADRs were lopinavir/ritonavir, hydroxychloroquine, and azithromycin.

The frequency of ADRs in patients treated during the first wave varies between studies, probably due to the diversity in both study methodology and the drugs used to treat the condition in different centers. Sun et al. described a 37.8% prevalence of ADRs, while Ramirez et al. reported an incidence of 760.63 (CI 95% 707.89–816.01) per 10,000 patients, in studies based on triggers to identify suspected serious ADRs [13,18]. In a random sample of patients admitted to US hospitals, 12% had ADRs, with a higher rate in those enrolled in clinical trials [3].

Diarrhea was the most frequent ADR in our study, representing nearly half of all ADRs, largely due to the high impact of lopinavir/ritonavir-induced diarrhea. As an adverse event, diarrhea has been described in approximately 50% of patients with human immunodeficiency virus infection during clinical trials with lopinavir/ritonavir [19]. Nonetheless, if we consider the total number of patients exposed to lopinavir/ritonavir in our registry, the frequency of diarrhea with this drug was lower (16.6%). Given the high frequency of this ADR and its low severity, it is possible that in some cases no special mention was made in medical records. Other explanations could include a lower incidence of diarrhea probably related to lopinavir/ritonavir in patients with COVID-19 infection (as the frequency of this adverse event in clinical trials in this population was lower, at 4.2%), or the possibility that it was considered to be a symptom related to the disease [20].

Most ADRs were previously known for the majority of drugs considered to have been possibly involved, although in some cases other drugs with a temporary relationship with the ADR were also considered suspicious. Only four ADRs were previously unknown. Alternative causes were difficult to assess given the novelty of the disease, along with the possibility that COVID-19 itself could explain some of the signs or symptoms registered as ADRs, including cases of well-known ADRs such as diarrhea in patients receiving lopinavir/ritonavir, as well as some cases of hepatitis. The inflammation–drug interaction in these patients has been highlighted as a plausible mechanism of injury to the liver (or other organs) [12,21].

The majority of ADRs resulted in recovery of the patient, although 7.1% of ADRs (in 14 patients) were serious, and 6 patients died. Except for one case of intestinal perforation with tocilizumab and two cases of hepatitis, serious cases corresponded to cardiac disorders with arrhythmias or sudden death otherwise unexplained, in which no previous electrocardiogram was available, and where the possibility of an arrhythmia secondary to COVID-19 could not be completely ruled out. It is well known that hydroxychloroquine, lopinavir/ritonavir, azithromycin, and other drugs may prolong the QTc interval and increase the risk of ventricular arrhythmias and drug-induced sudden death. Long QTc interval and arrhythmia are the primary manifestations of drug-induced cardiovascular injury caused by anti-COVID-19 drugs used during the first wave [6,7,8,9,10,11,22,23]. Bernardini et al. documented a prolonged QTc interval in 61% of COVID-19 patients treated with hydroxychloroquine alone or in combination with azithromycin, but only four (4%) patients showed a QTc > 500 ms, and no arrhythmic fatalities were described [24]. In another study with propensity-matching of groups, there were no differences in in-hospital mortality, life-threatening arrhythmias, or incidence of pulseless electrical activity arrest between a group of patients treated with hydroxychloroquine and azithromycin and untreated control groups, although QTc intervals were longer in patients receiving both treatments, and one patient developed drug-related ventricular tachycardia [9]. Once again, it is also challenging to distinguish mortality and cardiac arrhythmias due to COVID-19 from those related to its treatment [10,12].

As expected, patients with ADRs were more commonly treated with more drugs for COVID-19 infection than patients without ADRs. Regarding baseline characteristics, an increased risk of ADRs was seen in patients who were younger, taking less medication regularly, and with less presence of certain comorbidities in our study. This was probably related to the differences in the drugs used according to age, as well as the presence of some comorbidities, such as arterial hypertension. Lopinavir/ritonavir was the drug most commonly implicated as a suspect in ADRs, and it was used more in young patients and less in those with arterial hypertension. Sun et al., in their retrospective analysis in a hospital in China, identified the length of stay, number of drugs used in the hospital, and the presence of underlying basic diseases as independent risk factors for ADRs, with no differences regarding gender or age [13]. Zekarias et al. analyzed ADRs reported in VigiBase for drugs used in the treatment of COVID-19 in comparison with their use in other indications, specifically focusing on sex differences [25]. Their results revealed a male predominance of ADR reports for drugs used for COVID-19. Some gender differences in the reporting patterns need further elucidation—for example, the fact that hepatic and kidney-related events were mostly reported in males. We did not find differences in the risk of ADRs according to gender, but a specific analysis of the type of ADRs was not performed. On the other hand, the role of the disease itself, affecting males more severely, could be an important confounding factor.

The role of the disease is the principal limitation when assessing drug causality in suspected ADRs—especially during the first wave of COVID-19, when the disease was scarcely known. However, we were already aware of the disease as a possible cause of diarrhea, hepatitis, and systemic damage (other than the lung involvement) and, as with other studies [18], a great effort was made to carefully assess the temporary causality of drugs and the evolution of ADRs in order to better analyze the possible contribution of the drugs to the ADRs.

A strength of our study is that the register allowed us to identify a wide variety of ADRs suspected by clinicians attending patients. The design of the two largest studies previously reported was completely different, as the detection of possible ADRs was based on an active follow-up model with identification of triggers (biochemical or other) suggestive of ADRs [13,18]. On the other hand, we were able to carry out a causality assessment of drugs (patients’ medical records were reviewed if more information was needed), and to identify not only the most frequent ADRs, but also all of the drugs that were possibly involved.

The main limitation is that this is a retrospective observational study, in which information on ADRs was collected from a registry. Therefore, the incidence of ADRs could have been underestimated. However, serious effects are recorded in patients’ medical records. On the other hand, the study was carried out in only one center, so the external validity of the results may be limited. However, this is one of the public hospitals with the largest numbers of beds in Catalonia, with an extensive registry of patients, and it is to be expected that data can be extrapolated and are representative of patients treated in other hospitals with similar characteristics.

In conclusion, ADRs with treatments used during the first months of the COVID-19 pandemic were frequent, and consisted mainly of gastrointestinal, hepatobiliary, and cardiac disorders. Patients with ADRs were younger, taking less medication regularly, with a lower frequency of some comorbidities, and were more frequently exposed to multiple treatments. The drugs most frequently considered to be involved in ADRs were lopinavir/ritonavir, hydroxychloroquine, and azithromycin. Although most ADRs recovered without sequelae, fatal cases were described, although the role of the disease could not be completely ruled out. We now have results from clinical trials indicating that these treatments are not effective in patients with COVID-19. With the available information and looking back, in similar emergency situations, efforts should be made to use drugs in the context of clinical trials, and to limit off-label drug use to those drugs with a better benefit/risk profile in a specific situation, and for patients at risk of a worse disease prognosis.

## Figures and Tables

**Table 1:** Baseline characteristics of patients.

	All Patients (*n* = 933)	Patients with ADRs (*n* = 197)	Patients without ADRs (*n* = 736)	*p*
**Personal and clinical data; *n* (% of patients), unless otherwise specified**				
Age (years); median (IQR)	67.7 (52.1–78.8)	62.0 (50.7–73.2)	70.1 (53.2–80.1)	<0.001
Male	507 (54.3)	95 (48.2)	412 (56.0)	
Female	426 (45.7)	102 (51.8)	324 (44.0)	0.052
Pregnancy	14 (3.3)	4 (3.9)	10 (3.1)	0.750
Obesity (2 missing)	177 (19.0)	41 (20.9)	136 (18.5)	0.444
BMI (kg/m^2^); median (IQR)	33.5 (31.2–37)	33.8 (31.4–36.4)	33.4 (31.2–37.9)	0.567
History of drug allergy	143 (15.3)	34 (17.3)	109 (14.8)	0.397
ECOG prior to admission (grade 0)	725 (77.7)	162 (82.2)	563 (77.7)	0.086
Moderate/severe cognitive impairment	51 (5.5)	3 (1.5)	48 (6.5)	0.006
Active smoker	39 (4.2)	6 (3.0)	33 (4.5)	0.370
Active alcoholism	38 (4.1)	5 (2.5)	33 (4.5)	0.220
**Comorbidities; *n* (% of patients)**				
Hypertension	446 (47.8)	74 (37.4)	372 (50.5)	0.001
Diabetes	185 (19.8)	32 (16.2)	153 (20.8)	0.155
Chronic renal failure	115 (12.3)	20 (10.2)	95 (12.9)	0.296
Chronic respiratory disease	191 (20.5)	34 (17.3)	157 (21.3)	0.208
Heart failure	63 (6.8)	7 (3.6)	56 (7.6)	0.044
Ischemic heart disease	92 (9.9)	13 (6.6)	79 (10.7)	0.084
Atrial fibrillation	86 (9.2)	16 (8.1)	70 (9.5)	0.549
Immunosuppression	86 (9.2)	24 (12.2)	62 (8.4)	0.105
History of solid malignancy	112 (12.0)	25 (12.7)	87 (11.8)	0.739
History of leukemia/lymphoma	21 (2.3)	5 (2.5)	16 (2.2)	0.787
HCV/VHB infection	19 (2.0)	0	19 (2.6)	0.019
Non-viral liver disease	33 (3.5)	4 (2.0)	29 (3.9)	0.197
Cirrhosis	8 (0.9)	0	8 (1.1)	0.215
CNS disease	93 (10.0)	10 (5.1)	83 (11.3)	0.010
Psychiatric disorder	93 (10.0)	23 (11.7)	70 (9.5)	0.368
**Usual medication; n (% of patients)**				
Yes	706 (75.7)	137 (69.5)	569 (77.3)	0.031
Proton-pump inhibitors (A02BC)	323 (34.6)	57 (28.9)	266 (36.1)	0.059
HMG CoA reductase inhibitors (C10AA)	26.5 (247)	44 (22.3)	203 (27.6)	0.138
Anilides, analgesics (N02BE)	142 (15.2)	22 (11.2)	120 (16.3)	0.075
ACE inhibitors (C09AA)	133 (14.3)	27 (13.7)	106 (14.4)	0.804
Platelet aggregation inhibitors, excluding heparin (B01AC)	131 (14.0)	24 (12.2)	107 (14.5)	0.398
Beta-blocking agents, selective (C07AB)	122 (13.1)	22 (11.2)	100 (13.6)	0.371
Dihydropyridine derivatives (C08CA)	121 (13.0)	19 (9.6)	102 (13.9)	0.118
Sulfonamides, diuretics (C03CA)	113 (12.1)	23 (11.7)	90 (12.2)	0.833
Benzodiazepine derivatives (N05BA)	108 (11.6)	16 (8.1)	92 (12.5)	0.088
Angiotensin II receptor blockers (C09CA)	95 (10.2)	22 (11.2)	73 (9.9)	0.607
**COVID-19 data; *n* (% of patients)**				
Intensive care unit admission	118 (12.6)	32 (16.2)	86 (11.7)	0.087
Oxygen saturation (pulse oximeter); median (IQR)	96 (93–98)	96 (94–98)	96 (93–98)	0.479
Outcome: patient recovered from COVID-19	692 (74.2)	166 (84.3)	526 (71.5)	<0.001

ADRs: adverse drug reactions; IQR: interquartile range; BMI: body mass index; ECOG: Eastern Collaborative Group performance status; HCV: hepatitis C virus; HBV: hepatitis B virus; CNS: central nervous system.

**Table 2:** Numbers of drugs used for COVID-19 treatment or related complications.

Number of Drugs Per Patient	All Patients (*n* = 933) *n* (%)	Patients with ADRs (*n* = 197) *n* (%)	Patients without ADRs (*n* = 736) *n* (%)
1	35 (3.8)	2 (1.0)	33 (4.5)
2	95 (10.2)	15 (7.6)	80 (10.9)
3	163 (17.5)	23 (11.7)	140 (19.0)
4	377 (40.4)	81 (41.1)	296 (40.2)
5	156 (16.7)	44 (22.3)	112 (15.2)
≥6	107 (11.5)	32 (16.2)	75 (10.2)

**Table 3:** ADRs according to the affected system organ class.

System Organ Class Disorders	*n* (%)
Gastrointestinal disorders	161 (67.1)
Hepatobiliary disorders	26 (10.8)
Cardiac disorders	8 (3.3)
Investigations	7 (2.9)
Skin and subcutaneous tissue disorders	7 (2.9)
General disorders and administration site conditions	4 (1.7)
Renal and urinary disorders	4 (1.7)
Endocrine disorders	3 (1.3)
Metabolism and nutrition disorders	3 (1.3)
Nervous system disorders	3 (1.3)
Psychiatric disorders	3 (1.3)
Vascular disorders	3 (1.3)
Blood and lymphatic system disorders	2 (0.8)
Respiratory, thoracic, and mediastinal disorders	2 (0.8)
Ear and labyrinth disorders	1 (0.4)
Infections and infestations	1 (0.4)
Injury, poisoning, and procedural complications	1 (0.4)
Musculoskeletal and connective tissue disorders	1 (0.4)
Total	240 (100)

**Table 4:** Drugs deemed to be suspicious in ADRs.

Drugs	Administered Drugs ^a,b^	Patients with ADRs (*n* = 197)		
		*n*	% of That Drug Use	% of Total Patients with ADRs
Lopinavir/ritonavir	628	162	25.8	82.2
Hydroxychloroquine	840	142	16.9	72.1
Azithromycin	814	131	16.1	66.5
Ceftriaxone	663	89	13.4	45.2
Darunavir/cobicistat	166	19	11.4	9.6
Tocilizumab	174	17	9.8	8.6
Levofloxacin	35	11	31.4	5.6
Interferon beta-1b	47	11	23.4	5.6
Amoxicillin + clavulanic acid	41	7	17.1	3.5
Piperacillin/tazobactam	65	7	10.8	3.5
Prednisone	37	2	5.4	1.0
Methylprednisolone	92	2	2.2	1.0
Meropenem	38	1	2.6	0.5
Hydrocortisone	14	0	0	0
Dexamethasone	8	0	0	0

^a^ Number of patients with each drug (patients were exposed to at least one drug). ^b^ Others: acenocoumarol, enoxaparin, metoclopramide, micafungin, propofol, and tacrolimus, involved in 2 ADRs each; aztreonam, cefotaxime, ceftazidime, cotrimoxazole, digoxin, metamizole, nitroglycerin, quetiapine, risperidone, salbutamol, and valsartan, involved in 1 ADR each.

**Table 5:** Age of the patients and percentage of patients with arterial hypertension according to the drugs administered.

Drug	Median Age of Patients (Years)			% of Patients with Arterial Hypertension		
	Administered Drug			Administered Drug		
	Yes	No	*p*	Yes	No	*p*
Hydroxychloroquine	66.79	75.36	0.001	47.38	51.61	0.438
Azithromycin	67.61	67.95	0.668	47.91	47.06	0.862
Ceftriaxone	69.46	63.20	0.026	50.53	41.11	0.009
Lopinavir/ritonavir	62.05	76.23	<0.001	41.08	61.64	<0.001
Tocilizumab	61.95	69.46	<0.001	40.80	49.41	0.041
Darunavir/cobicistat	73.83	66.03	<0.001	59.04	45.37	0.001
Methylprednisolone	70.96	67.21	0.172	50.00	47.56	0.657
Piperacillin/tazobactam	65.81	68.23	0.335	53.85	47.35	0.312
Others	60.49	68.46	0.027	33.33	48.69	0.028
Interferon beta-1b	62.46	68.38	0.035	42.55	48.08	0.460
Amoxicillin + clavulanic acid	71.22	67.42	0.768	39.02	48.21	0.250
Meropenem	58.59	68.47	0.006	44.74	47.93	0.699
Prednisone	68.68	67.61	0.588	78.38	46.54	<0.001
Levofloxacin	74.26	67.41	0.058	57.14	47.44	0.260
Hydrocortisone	64.83	67.89	0.378	42.86	47.88	0.709
Dexamethasone	62.45	67.89	0.393	12.50	48.11	0.071

**Table 6:** Principal ADRs and suspicious drugs ^a^.

ADR, *n* (%)	Suspicious Drugs in ≥2 ADRs
Diarrhea, 113 (47.1%)	Lopinavir/ritonavir (104), hydroxychloroquine (79), azithromycin (75), ceftriaxone (57), darunavir/cobicistat (8), tocilizumab (6), amoxicillin/clavulanic acid (4), piperacillin/tazobactam (3), levofloxacin (2)
Nausea, 22 (9.2%)	Lopinavir/ritonavir (18), hydroxychloroquine (17), azithromycin (13), ceftriaxone (11), darunavir/cobicistat (3), levofloxacin (2)
Vomiting, 17 (7.1%)	Azithromycin (15), lopinavir/ritonavir (15), hydroxychloroquine (13), ceftriaxone (10)
Hyperbilirubinemia, 10 (4.2%)	Lopinavir/ritonavir (10), azithromycin (8), hydroxychloroquine (8), interferon beta-1b (4), ceftriaxone (3), levofloxacin (3), tocilizumab (2)
Electrocardiogram QT prolonged, 7 (2.9%)	Azithromycin (6), hydroxychloroquine (5), lopinavir/ritonavir (3), ceftriaxone (2)
Mixed liver injury, 6 (2.5%)	Azithromycin (4), hydroxychloroquine (4), lopinavir/ritonavir (4), ceftriaxone (2), interferon beta-1b (2), piperacillin/tazobactam (2), tocilizumab (2)
Hepatic cytolysis, 5 (2.1%)	Azithromycin (5), hydroxychloroquine (5), lopinavir/ritonavir (5), ceftriaxone (3), tocilizumab (3)
Acute kidney injury, 4 (1.7%)	Hydroxychloroquine (4), azithromycin (3), lopinavir/ritonavir (3)
Sudden death, 4 (1.7%)	Hydroxychloroquine (4), azithromycin (3), lopinavir/ritonavir (3), levofloxacin (2)
Other, 52 (21.7%)	

^a^ More than one drug could be involved in one ARD. Complete information can be found in the Supplementary Material.

## Data Availability

The data presented in this study are available upon request from the corresponding author.

## Supplementary Materials

The following supporting information can be downloaded at: https://www.mdpi.com/article/10.3390/diagnostics12071612/s1, Figure S1: Differences in the age of patients according to the administration of the drugs; Figure S2: Differences in the percentage of patients with arterial hypertension according to the administration of the drugs; Table S1: ADRs and suspicious drugs.

## References

[1] McCreary E.K., Pogue J.M. (2020). Coronavirus Disease 2019 Treatment: A Review of Early and Emerging Options. Open Forum Infect. Dis..

[2] Spanish Agency for Medicine and Health Products Tratamientos Disponibles Para el Manejo de la Infección Respiratoria Por SARS-CoV-2 [Treatments Available for the Management of Respiratory Infection by SARS-CoV-2]. https://www.aemps.gob.es/laAEMPS/docs/medicamentos-disponibles-SARS-CoV-2-16-4-2020.pdf?x35385.

[3] Rhodes N.J., Dairem A., Moore W.J., Shah A., Postelnick M.J., Badowski M.E., Michienzi S.M., Borkowski J.L., Polisetty R.S., Fong K. (2021). Multicenter point prevalence evaluation of the utilization and safety of drug therapies for COVID-19 at the onset of the pandemic timeline in the United States. Am. J. Health Syst. Pharm..

[4] Spain, Ministry of Health and Social Policy Real Decreto 1015/2009, de 19 de Junio, Por el Que se Regula la Disponibilidad de Medicamentos en Situaciones Especiales [Royal Decree 1015/2009, of June 19, Which Regulates the Availability of Medicines in Special Situations]. https://www.boe.es/boe/dias/2009/07/20/pdfs/BOE-A-2009-12002.pdf.

[5] Crescioli G., Brilli V., Lanzi C., Burgalassi A., Ieri A., Bonaiuti R., Romano E., Innocenti R., Mannaioni G., Vannacci A. (2021). Adverse drug reactions in SARS-CoV-2 hospitalised patients: A case-series with a focus on drug-drug interactions. Intern Emerg. Med..

[6] Chatre C., Roubille F., Vernhet H., Jorgensen C., Pers Y.M. (2018). Cardiac complications attributed to chloroquine and hydroxychloroquine: A systematic review of the literature. Drug Saf..

[7] Chorin E., Wadhwani L., Magnani S., Dai M., Shulman E., Nadeau-Routhier C., Knotts R., Bar-Cohen R., Kogan E., Barbhaiya C. (2020). QT interval prolongation and torsade de pointes in patients with COVID-19 treated with hydroxychloroquine/azithromycin. Heart Rhythm.

[8] Gérard A., Romani S., Fresse A., Viard D., Parassol N., Granvuillemin A., Chouchana L., Rocher F., Drici M.-D., French Network of Pharmacovigilance Centers (2020). “Off-label” use of hydroxychloroquine, azithromycin, lopinavir-ritonavir and chloroquine in COVID-19: A survey of cardiac adverse drug reactions by the French Network of Pharmacovigilance Centers. Therapies.

[9] Huang H.D., Jneid H., Aziz M., Ravi V., Sharma P.S., Larsen T., Chatterjee N., Saour B., Aziz Z., Nayak H. (2020). Safety and effectiveness of hydroxychloroquine and azithromycin combination therapy for treatment of hospitalized patients with COVID-19: A propensity-matched study. Cardiol. Ther..

[10] Kochav S.M., Coromilas E., Nalbandian A., Ranard L.S., Gupta A., Chung M.K., Gopinathannair R., Biviano A.B., Garan H., Wan E.Y. (2020). Cardiac arrhythmias in COVID-19 infection. Circ. Arrhythm. Electrophysiol..

[11] Rosenberg E.S., Dufort E.M., Udo T., Wilberschied L.A., Kumar J., Tesoriero J., Weinberg P., Kirkwood J., Muse A., DeHovitz J. (2020). Association of treatment with hydroxychloroquine or azithromycin with in-hospital mortality in patients with COVID-19 in New York State. JAMA.

[12] Ommati M.M., Mobasheri A., Heidari R. (2021). Drug-induced organ injury in coronavirus disease 2019 pharmacotherapy: Mechanisms and challenges in differential diagnosis and potential protective strategies. J. Biochem. Mol. Toxicol..

[13] Sun J., Deng X., Chen X., Huang J., Huang S., Li Y., Feng J., Liu J., He G. (2020). Incidence of Adverse Drug Reactions in COVID-19 patients in China: An active monitoring study by Hospital Pharmacovigilance System. Clin. Pharmacol. Ther..

[14] Medical Dictionary for Regulatory Activities. https://www.meddra.org/.

[15] (2021). WHO Collaborating Centre for Drug Statistics Methodology, ATC Classification Index with DDDs. https://www.whocc.no/atc_ddd_index/.

[16] Aguirre C., García M. (2016). Evaluación de la causalidad en las comunicaciones de reacciones adversas a medicamentos. Algoritmo del Sistema Español de Farmacovigilancia [Causality assessment in reports on adverse drug reactions. Algorithm of Spanish pharmacovigilance system]. Med. Clin..

[17] (2004). CPMP/ICH/3945/03; Clinical Safety Data Management: Definitions and Standards for Expedited Reporting. European Medicines Agency: Amsterdam, The Netherlands.

[18] Ramírez E., Urroz M., Rodríguez A., González-Muñoz M., Martín-Vega A., Villán Y., Seco E., Monserrat J., Frías J., Carcas A.J. (2020). Incidence of suspected serious adverse drug reactions in Corona Virus Disease-19 patients detected by a pharmacovigilance program by laboratory signals in a tertiary hospital in Spain: Cautionary data. Front. Pharmacol..

[19] European Medicines Agency (2006). Kaletra: EPAR-Scientific Discussion. https://www.ema.europa.eu/en/documents/scientific-discussion/kaletra-epar-scientific-discussion_en.pdf.

[20] Cao B., Wang Y., Wen D., Liu W., Wang J., Fan G., Ruan L., Song B., Cai Y., Wei M. (2020). A trial of lopinavir-ritonavir in adults hospitalized with severe COVID-19. N. Engl. J. Med..

[21] Olry A., Meunier L., Délire B., Larrey D., Horsmans Y., Le Louët H. (2020). Drug-induced liver injury and COVID-19 infection: The rules remain the same. Drug. Saf..

[22] Grandvuillemin A., Drici M.D., Jonville-Bera A.P., Micallef J., Montastruc J.L., French Pharmacovigilance Network (2021). French pharmacovigilance public system and COVID-19 pandemic. Drug. Saf..

[23] Skalafouris C., Samer C., Stirnemann J., Grosgurin O., Eggimann F., Grauser D., Reny J., Bonnabry P., Guignard B. (2021). Electronic monitoring of potential adverse drug events related to lopinavir/ritonavir and hydroxychloroquine during the first wave of COVID-19. Eur. J. Hosp. Pharm..

[24] Bernardini A., Ciconte G., Negro G., Rondine R., Mecarocci V., Viva T., Santini F., Innocentiis C., Giannelli L., Witkowska E. (2021). Assessing QT interval in COVID-19 patients: Safety of hydroxychloroquine-azithromycin combination regimen. Int. J. Cardiol..

[25] Zekarias A., Watson S., Hedfors Vidlin S., Grundmark B. (2020). Sex differences in reported adverse drug reactions to COVID-19 drugs in a global database of individual case safety reports. Drug Saf..

