# Supplementary material for: Safety of Drugs Used during the First Wave of COVID-19: A Hospital-Registry-Based Study
Source: Diagnostics (Basel). 2022 Jul 1;12(7):1612. doi: 10.3390/diagnostics12071612 (PMC9316110; doi:10.3390/diagnostics12071612)
Supplement: Supplementary file 1 [file diagnostics-12-01612-s001.zip › diagnostics-1691814-supplementary.pdf]

Supplementary material
Safety of drugs used during the first COVID-19 wave: a hospital registry-based study

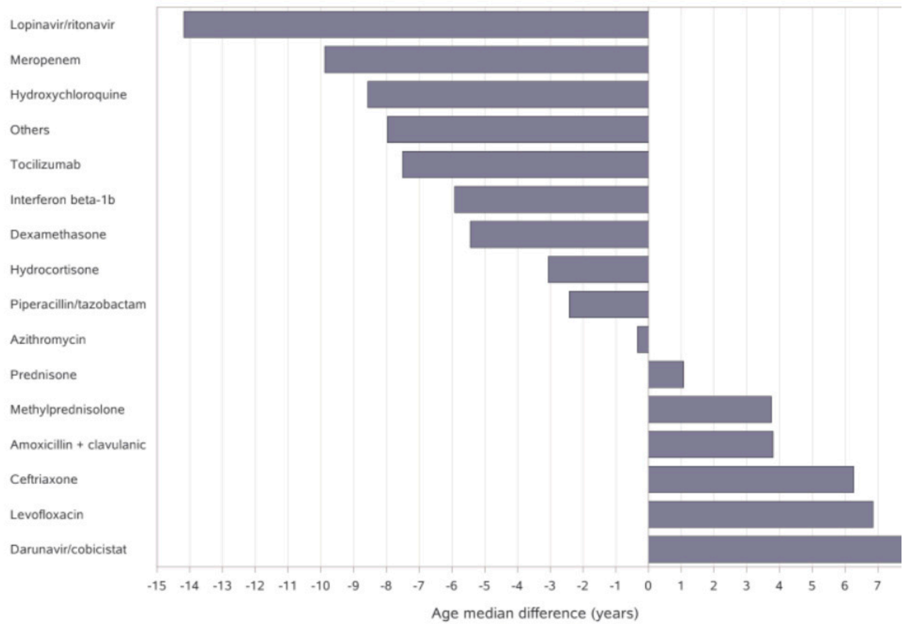

Figure S1. Difference in the age of patients according to the administration of the drugs.

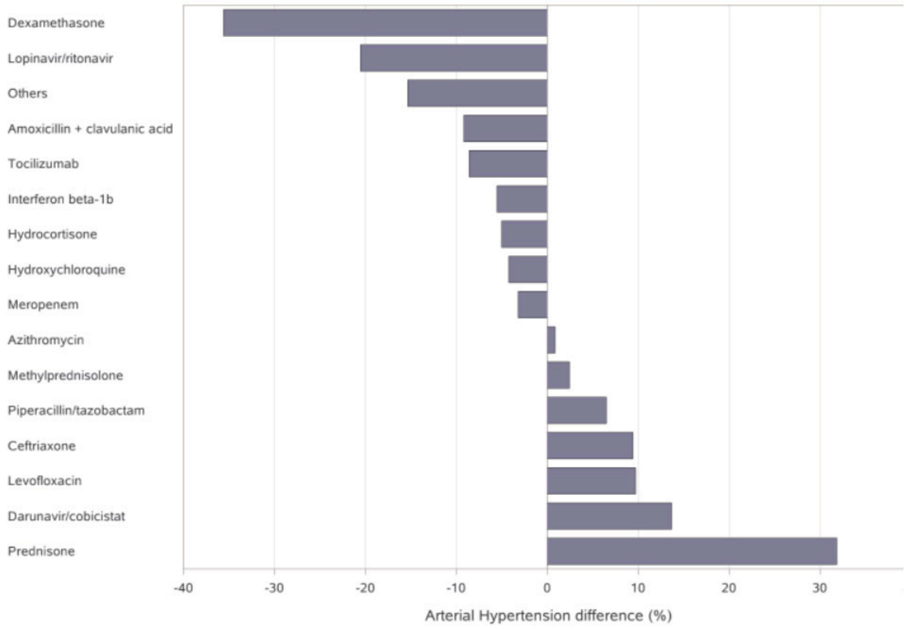

Figure S2. Difference in the percentage of patients with arterial hypertension according to the administration of the drugs.

Table S1. ADRs and suspicious drugs.

ADR	Suspicious drugs
**Gastrointestinal disorders (n=161)**	
Diarrhea (n=113)	lopinavir/ritonavir (104), hydroxychloroquine (79), azithromycin (75), ceftriaxone (57), darunavir/cobicistat (8), tocilizumab (6), amoxicillin/clavulanic acid (4), piperacillin/tazobactam (3), levofloxacin (2), interferon beta-1b (1), micafungin (1)
Nausea (n=22)	lopinavir/ritonavir (18), hydroxychloroquine (17), azithromycin (13), ceftriaxone (11), darunavir/cobicistat (3), levofloxacin (2), amoxicillin/clavulanic acid (1)
Vomiting (n=17)	azithromycin (15), lopinavir/ritonavir (15), hydroxychloroquine (13), ceftriaxone (10), darunavir/cobicistat (1), interferon beta-1b (1)
Abdominal pain (n=2)	azithromycin (2), hydroxychloroquine (2), lopinavir/ritonavir (2), ceftriaxone (1),
Dyspepsia (n=2)	hydroxychloroquine (2), lopinavir/ritonavir (2), azithromycin (1), ceftriaxone (1), darunavir-cobicistat (1),
Abdominal pain upper (n=1)	azithromycin, ceftriaxone, hydroxychloroquine, lopinavir/ritonavir
Constipation (n=1)	lopinavir/ritonavir
Dysphagia (n=1)	darunavir/cobicistat, hydroxychloroquine, lopinavir/ritonavir
Large intestine perforation (n=1)	tocilizumab
Mouth haemorrhage (n=1)	enoxaparin
**Hepatobiliary disorders (n=26)**	
Hyperbilirubinemia (n=10)	lopinavir/ritonavir (10), azithromycin (8), hydroxychloroquine (8), interferon beta-1b (4), ceftriaxone (3), levofloxacin (3), tocilizumab (2), cefotaxime (1), piperacillin/tazobactam (1), propofol (1)
Mixed liver injury (n=6)	azithromycin (4), hydroxychloroquine (4), lopinavir/ritonavir (4), ceftriaxone (2), interferon beta-1b (2), piperacillin/tazobactam (2), tocilizumab (2), cotrimoxazole (1), darunavir/cobicistat (1), metoclopramide (1), micafungin (1), prednisone (1), tacrolimus (1)
Hepatic cytolysis (n=5)	azithromycin (5), hydroxychloroquine (5), lopinavir/ritonavir (5), ceftriaxone (3), tocilizumab (3), amoxicillin/clavulanic acid (1), interferon beta-1b (1), levofloxacin (1), piperacillin/tazobactam (1)
Hepatitis cholestatic (n=3)	azithromycin (3), ceftriaxone (3), hydroxychloroquine (2), interferon beta-1b (2), lopinavir/ritonavir (2), amoxicillin/clavulanic (1), darunavir/cobicistat (1)
Hypertransaminasemia (n=2)	lopinavir/ritonavir (2), azithromycin (1), ceftriaxone (1), hydroxychloroquine (1), propofol (1)
**Cardiac disorders (n=8)**	
Bradycardia (n=2)	azithromycin (2), hydroxychloroquine (2), lopinavir/ritonavir (2), tocilizumab (1)
Atrial fibrillation (n=1)	Azithromycin, ceftriaxone, darunavir/cobicistat, hydroxychloroquine
Atrial flutter (n=1)	Azithromycin, ceftriaxone, darunavir/cobicistat, hydroxychloroquine, interferon beta-1b, lopinavir/ritonavir
Cardiac arrest (n=1)	digoxin, hydroxychloroquine, metamizole, nitroglycerin
Cardiogenic shock (n=1)	azithromycin, hydroxychloroquine

Supraventricular tachyarrhythmia (n=1)	azithromycin, hydroxychloroquine
Tachycardia (n=1)	digoxin, hydroxychloroquine, metamizole, nitroglycerin
**Investigations (n=7)**	
Electrocardiogram QT prolonged (n=7)	azithromycin (6), hydroxychloroquine (5), lopinavir/ritonavir (3), ceftriaxone (2), metoclopramide (1), propofol (1), salbutamol (1), tocilizumab (1)
**Skin and subcutaneous tissue disorders (n=7)**	
Rash (n=3)	azithromycin (2), hydroxychloroquine (2), lopinavir/ritonavir (2), amoxicillin/clavulanic (1), aztreonam (1), ceftriaxone (1), interferon beta-1b (1), meropenem (1)
Rash maculo-papular (n=2)	azithromycin (1), ceftriaxone (1), hydroxychloroquine (1), levofloxacin (1), lopinavir/ritonavir (1)
Rash erythematous (n=1)	azithromycin, ceftriaxone, hydroxychloroquine, lopinavir/ritonavir
Urticaria (n=1)	azithromycin, ceftriaxone, hydroxychloroquine, darunavir/cobicistat
**General disorders and administration site conditions (n=4)**	
Sudden death (n=4)	hydroxychloroquine (4), azithromycin (3), lopinavir/ritonavir (3), levofloxacin (2), metoclopramide (1), propofol (1), quetiapine (1), risperidone (1), salbutamol (1)
**Nervous system disorders (n=3)**	
Dizziness (n=2)	darunavir/cobicistat (1), lopinavir/ritonavir (1)
Headache (n=1)	azithromycin (1), ceftriaxone (1), hydroxychloroquine (1), lopinavir/ritonavir (1)
**Renal and urinary disorders (n=4)**	
Acute kidney injury (n=4)	hydroxychloroquine (4), azithromycin (3), lopinavir/ritonavir (3), ceftazidime (1), ceftriaxone (1), piperacillin/tazobactam (1), tacrolimus (1)
**Endocrine disorders (n=3)**	
Hyperglycemia (n=2)	methylprednisolone (2), ceftriaxone (1), darunavir/cobicistat (1)
Impaired fasting glucose (n=1)	prednisone (1)
**Metabolism and nutrition disorders (n=3)**	
Anorexia (n=1)	azithromycin (1), ceftriaxone (1), darunavir/cobicistat (1), hydroxychloroquine (1), lopinavir/ritonavir (1)
Hypertriglyceridemia (n=2)	azithromycin (2), ceftriaxone (2), hydroxychloroquine (2), lopinavir/ritonavir (2), tocilizumab (2)
**Psychiatric disorders (n=3)**	
Agitation (n=1)	azithromycin (1), ceftriaxone (1), interferon beta-1b (1), lopinavir/ritonavir (1)
Confusion (n=1)	azitromicina (1), ceftriaxone (1), interferon beta-1b (1), lopinavir/ritonavir (1)
Delirium (n=1)	azithromycin (1), hydroxychloroquine (1), tocilizumab (1), metoclopramide (1), prednisone (1), tacrolimus (1)
**Vascular disorders (n=3)**	
Chest wall hematoma (n=1)	acenocoumarol (1)

Hypotension (n=2)	hydroxychloroquine (2), azithromycin (1), ceftriaxone (1), digoxin (1), lopinavir/ritonavir (1), metamizole (1), nitroglycerin (1), valsartan (1)
**Blood and lymphatic system disorders (n=2)**	
Hypofibrinogenemia (n=1)	tocilizumab (1)
Leukopenia (n=1)	tocilizumab (1), azithromycin (1), hydroxychloroquine (1), interferon beta-1b (1), lopinavir/ritonavir (1)
**Respiratory, thoracic and mediastinal disorders (n=2)**	
Epistaxis (n=2)	acenocoumarol (1), darunavir/cobicistat (1), hydroxychloroquine (1), enoxaparin (1)
**Ear and labyrinth disorders (n=1)**	
Hypoacusis (n=1)	azithromycin (1), hydroxychloroquine (1)
**Infections and infestations (n=1)**	
Septic shock (n=1)	tocilizumab (1)
**Injury, poisoning and procedural complications (n=1)**	
Fall (n=1)	azithromycin (1), ceftriaxone (1), interferon beta-1b (1), lopinavir/ritonavir (1)
**Musculoskeletal and connective tissue disorders (n=1)**	
Myopathy (n=1)	hydroxychloroquine (1)
